# Metabolomic and Lipidomic Approaches to Evaluate the Effects of *Eucommia ulmoides* Leaves on Milk Quality and Biochemical Properties

**DOI:** 10.3389/fvets.2021.644967

**Published:** 2021-06-01

**Authors:** Zhanwei Teng, Linfeng Wang, Hongyan Du, Gaiqing Yang, Tong Fu, Hongxia Lian, Yu Sun, Shenhe Liu, Liyang Zhang, Tengyun Gao

**Affiliations:** ^1^College of Animal Science and Technology, Henan Agricultural University, Zhengzhou, China; ^2^Economic Forestry Research and Development Centre, Chinese Academy of Forestry Sciences, Zhengzhou, China; ^3^Modern Experimental Technique and Management Centre, Henan Agricultural University, Zhengzhou, China

**Keywords:** dairy cows, *Eucommia ulmoides* leaves, milk composition, milk metabolomics, milk lipidomics

## Abstract

*Eucommia ulmoides* leaves (EUL) contain a variety of natural bioactive compounds including chlorogenic acid, geniposide acid, and aucubin. These bioactive chemicals improve immune function and regulate lipid metabolism. The aim of this study was to investigate the effects of EUL on the biochemical properties of milk. Twenty Holstein dairy cows were randomly allocated to two groups fed a control (CTR, diet without EUL, *n* = 10) or EUL (diet containing 3% EUL, dry matter, *n* = 10) diet for 55 d. At the end of the experimental period (d 55), milk samples were collected and analyzed to determine their composition. Though levels of milk fat, protein, lactose, and total milk solids were similar between the groups, small molecules, metabolites, lipids, and cytokines differed. Compared with the CTR group, the EUL group had an improved cluster of differentiation (CD)4/CD8 ratio (*P* < 0.05) and lower interleukin (IL)-8 and IL-6 content (*P* < 0.05). Metabolomics analysis identified 14 metabolites including 7Z, 10Z, 13Z, 16Z, 19Z-docosapentaenoic acid (FC = 3.129), adrenic acid (FC = 2.830), and eicosapentaenoic acid (FC=1.685) as having significantly increased in the EUL group (*P* < 0.05) while 11 metabolites, including indole-2-carboxylic acid (FC = 0.636), cholic acid (FC = 0.430), and creatine (FC = 0.784) had significantly decreased (*P* < 0.05). Based on a constructed metabolome map, linoleic acid metabolism had the highest impact value for EUL. A total of 21 lipid classes and 1,094 lipid species were detected in the milk by lipidomic analysis, among which 40 differed significantly between the CTR and EUL groups. The present findings showed that the EUL altered milk composition. Correlation analysis showed that 7Z, 10Z, 13Z, 16Z, 19Z-docosapentaenoic acid, adrenic acid, and eicosapentaenoic acid levels were negatively correlated with those of the inflammatory factors IL-6 and IL-8 (*P* < 0.05), indicating that EUL improved milk quality by reducing inflammatory factors and increasing the CD4/CD8 ratio. Overall, our data demonstrate that EUL had positive effects on milk antioxidant parameters, immune indices, and micro-composition metabolism, thereby improving milk quality.

## Introduction

*Eucommia ulmoides* (Du-Zhong) is a well-known medical plant that has been used in Chinese medicine and food production since ancient times. In addition, *Eucommia ulmoides* has also been planted in Japan, Korea, and some other countries as a source for health drinks. *Eucommia ulmoides* leaves (EUL) are a facultative nutrient food source that contains natural bioactive compounds with multiple functions for healthcare (1). EUL contains more than 50 bioactive substances including chlorogenic acid, geniposide acid, and aucubin, which have antioxidant properties and improve immune function, as well as regulating lipid metabolism (2, 3). Supplementation with chlorogenic acid extract from EUL is reported to alleviate the negative effects of heat stress on meat quality and fatty acid profiles in broiler breast meat or mutton, by improving antioxidant status (4, 5). Additionally, fatty acid, amino acids, and polysaccharides were identified from EUL (6, 7). Zhu and Sun reported that EUL has rich polyunsaturated fatty acids (PUFA) such as linolelaidic acid and α-linolenic acid (7). Our previous research found that EUL can regulate lipid partitioning in sheep and improve mutton quality, as well as modulate glucose metabolism (8, 9). Therefore, raw EUL can be used as a high-quality animal foodstuff, or functional additive, for livestock.

Bovine milk is an important food source because milk contains all the essential nutrients for young people and other mammals (10); however, it is a mutable product that can be affected by many factors including diet, the environment, and physiological status. Recent studies have shown that forage quality has an important impact on the quality of milk. It was found that feeding low quality forage to cows not only exhibited low feed efficiency but also led to lower milk performance (11). The Chinese dairy industry suffers from limitations in forage quality. Until now, limited evidence has been available on the utilization of EUL to improve dairy cow milk composition and its biochemical properties. Therefore, understanding biochemical properties and metabolic mechanisms involved in milk production in response to EUL is helpful for developing strategies for effective, efficient utilization of the abundant EUL.

It is difficult to discern metabolic alterations of milk from cows fed specific diets using traditional measurement methods. Metabolomics and lipidomics analysis are effective approaches for studying metabolites in fluids and tissues, and other physiological and biochemical properties (12, 13). Therefore, in this study, metabolomics and lipidomics were used to assess the milk composition and metabolite profile of dairy cows in response to EUL, with the aim of evidencing its function on dairy cow metabolism.

## Materials and Methods

### Animals, Diets, and Experimental Design

All procedures were approved by the Animal Care and Use Committee of Henan Agricultural University (approval number: HENAU-2018-015). **Twenty** Holstein dairy cows of similar age (parity 2), lactation stage (150 ± 20 d), and milk production (25.2 ± 5.00 kg/d) were selected and randomly divided into two groups: CTR (control, diet without EUL, *n* = 10) and EUL (diet containing 3% EUL, dry matter, *n* = 10). The range of body condition scores of cows was 3.0–3.5. Before the formal experiment, we collected milk samples of experimental cows for milk composition analysis and found that there was no difference in milk composition before the EUL treatment (Supplementary Table 1). All the animals remained in good health throughout the experimental period with no visible deleterious health effects. This level of EUL inclusion (3%) in the diet was chosen based on the biochemical indicators, hormone concentrations, and immune parameters in blood results from preliminary studies (Supplementary Table 2). *Eucommia* trees were cultivated in Henan (34 39' N, 112 24' E). EUL were collected from *Eucommia* trees from September to October. After drying at 65°C for 72 h, EUL were crushed through a 40 mesh sieve to generate EUL powder, which was packaged and stored in waterproof bags until use. The composition and nutritional values of the EUL were as follows (dry matter basis, %): dry matter: 89.5%, crude protein: 8.3%, Ca: 2.06%, P: 0.15%, ether extract: 7.54% and its fatty acid composition is palmitic acid: 10.80%, hexadecatrienoic acid: 0.85%, linoleic acid: 1.95%, and linolenic acid: 45.58%. Geniposide acid, chlorogenic acid, and aucubin in EUL were quantified by high-performance liquid chromatography/tandem mass spectrometry (HPLC-MS/MS) (ProNetsBio, Wuhan, China) using a Poroshell 120 SB-C18 reverse phase column (2.1 ×150, 2.7 um; AGLIENT, United States). The geniposide acid, aucubin, and chlorogenic acid content in EUL were 77.15 ± 8.94, 20.71 ± 0.82, and 2,264.16 ± 46.51 μg/g, respectively. EUL was supplemented to the diet according to weight and fed as a mixed ratio. The diet was fed to the cows twice daily (at 7:00 and 17:00 h), with *ad libitum* intake. During the experiment, feed intake was recorded daily allowing for approximately 5–10% orts. The detailed composition and nutrient content of the diets are presented in Table 1.

**Table 1:** Experimental diet constituents.

**Item**	**CTR**	**EUL**
**Ingredient (%, DM basis)**		
Corn silage	25	25
Alfalfa hay	20	20
Oat hay	7	4
*Eucommia ulmoides* leaves	0	3
Steam-flaked corn	20	20
Cottonseed	3	3
Soybean meal	12.7	12.7
Beet pulp	2.1	2.1
Brewers grain	3.9	3.9
Molasses	2	2
Premix^a^	4.3	4.3
**Nutrient composition**		
DM, %	66.28	66.42
CP, % of DM	15.06	15.09
NDF, % of DM	31.74	31.04
ADF, % of DM	20.68	20.49
NFC, % of DM	44.15	44.61
Ca, % of DM	0.59	0.63
P, % of DM	0.33	0.33
NEL, Mcal/kg of DM	1.56	1.57

a *Formula to provide (per kg of DM) 500,000–700,000 IU of vitamin A, 140,000–170,000 IU of vitamin D3, 2,000–4,000 IU of vitamin E, 7,000–9,000 mg of Zn, 40–80 mg of Se, 180 mg of I, 1,400–2,500 mg of Fe, 15–30 mg of Co, 1,400–2,500 mg of Mn, and 1,400–2,500 mg of Cu.*

*CTR, basal diet; EUL, basal diet + 3% Eucommia ulmoides leaves; DM, dry matter; CP, crude protein; NDF, neutral detergent fiber; ADF, acid detergent fiber; NFC, non-fiber carbohydrates; NEL, net energy of lactation*.

Cows were housed in a shaded open barn, with free access to water. The trial duration was 55 d. Cows were gradually adjusted to the experimental diet over a 14 d transition period. In the experiment, milk production was determined three consecutive days per week using milk sampling devices (Waikato Milking Systems NZ Ltd., Waikato, Hamilton, New Zealand).

### Sampling and Analysis

At the end of the trial, two 50 mL aliquot milk samples of individual cows were collected proportional to the yield (4:3:3, composite) in the morning, noon, and evening. Regular parameters, including fat, protein, lactose, total milk solids, and somatic cell count (SCC), were analyzed using a MilkoScan FT^+^ instrument (Foss Electric A/S, Foss, Hillerod, Denmark). Samples allocated for metabolomics and lipidomics were immediately stored in a liquid nitrogen container to minimize any possible metabolite degradation, then transferred to a cryogenic refrigerator and stored at −80°C until further analysis.

For biochemical analysis, 10 mL of the milk samples were skimmed by centrifugation at 3,000 *g* for 15 min at 4°C, and then centrifuged at 12,000 *g* for 30 min at 4°C using a high-speed freezing centrifuge (Avanti JXN-30, Beckman Coulter, California, USA). Supernatants were separated into several clean vials to measure biochemical indices, related hormones, and immune indicators. Biochemical parameters, including superoxide dismutase (SOD), malondialdehyde (MDA), and glutathione peroxidase (GSH-Px); hormones, such as growth hormone (GH), triiodothyronine (T3), insulin (INS), glucagon (GC), cortisol (CORT), and adrenaline (ADR); and immune indicators, including immunoglobulin A (IgA), immunoglobulin G (IgG), tumor necrosis factor-α (TNF-α), interleukin-1β (IL-1β), interleukin-6 (IL-6), interleukin-8 (IL-8), interferon-γ (IFN-γ), cluster of differentiation 4 (CD4), and cluster of differentiation 8 (CD8), among others, were tested using ELISA kits (Shanghai BlueGene Biotechnology Co., Ltd., Shanghai, China), according to the manufacturer's instructions. The inter- and intra-assay coefficient of variations of these kits were <12 and <10%, respectively. These kits had no obvious cross-reactivity.

### Metabolomics

#### Sample Preparation and Extraction

Samples were thawed at 4°C and then shaken and mixed evenly. Milk samples (100 μL) were added to 400 μL of cold methanol/acetonitrile (1:1, v/v), and the mixture vortexed. Subsequently, all sample mixtures were stored at −20°C for 60 min, and then centrifuged at 14,000 *g* for 20 min at 4°C. Supernatants were collected, vacuum dried, and reconstituted with 100 μL water/acetonitrile (1:1, v/v). The reconstituted solution was vortexed and centrifuged at 14,000 *g* for 15 min at 4°C and the supernatant used for LC-MS/MS analysis.

#### Liquid Chromatography Positive Ion Electrospray Ionization Tandem Mass Spectrometry (LC-ESI-MS/MS) Analysis

Prepared samples were separated using a UHPLC HILIC column (1.7 μm, 2.1 mm ×100 mm; Waters, Massachusetts, United States). Column temperature was 25°C and the injection volume was 2 μL. Mobile phase A comprised 25 mM ammonium acetate and 25 mM aqua ammonia, and mobile phase B was acetonitrile. To prevent the effects of fluctuations in instrument signal detection, a random sequence was used for continuous analysis of the samples. Processed sample stability was determined by repeated analysis of quality control milk samples. During the entire process of analysis, samples were placed in an automatic sampler at 4°C.

Samples separated by UHPLC were analyzed on a Triple TOF 5600 mass spectrometer (AB SCIEX, Boston, United States). ESI positive and negative modes were used and reaction monitoring parameters were as follows: ion source gas 1, 60 psi; ion source gas 2, 60 psi; curtain gas (CUR), 30 psi; source temperature, 600°C, ion spray voltage floating, ± 5,500 V (positive and negative modes); time-of-flight mass spectrometry (TOF MS) scan m/z range, 60–1,000 Da; product ion scan m/z range, 25–1,000 Da; TOF MS scan accumulation time, 0.20 s/spectra; and product ion scan accumulation time, 0.05 s/spectra. Information-dependent acquisition (IDA) was used for secondary mass spectrometry, and a high-sensitivity model was adopted. The de-clustering potential was ± 60 V (positive and negative modes), with collision energy 35 ± 15 eV. The IDA parameters were as follows: exclude isotopes within 4 Da; candidate ions to monitor per cycle, 6.

### Lipidomics

#### Sample Preparation and Extraction

The samples were thawed slowly at 4°C and thoroughly vortex mixed. Milk samples (100 μL) were added to 200 μL water and vortexed. Cold methanol (200 μL) was then added to the composite milk samples and re-vortexed. The composite milk samples were then added to 800 μL methyl tert butyl ether and thoroughly vortex mixed. All sample mixtures were placed in an ultrasound water bath at 4°C for 20 min, and stored at 25°C for 30 min, before being centrifuged at 14,000 *g* for 15 min at 10°C. The supernatant was then decanted and freeze-dried in nitrogen and reconstituted with 300 μL 90% isopropanol/acetonitrile (1:1, v/v). The reconstituted solution was vortexed and centrifuged (14,000 *g*, for 15 min at 10°C). The resulting supernatant was used for LC-MS/MS analysis.

#### LC-ESI-MS/MS Analysis

Milk samples were separated using a Nexera LC-30A (Shimadzu, Kyoto, Japan) ultrahigh performance liquid chromatography (UHPLC) HILIC column (ACQUITY UPLC BEH Amide 1.7 μm, 2.1 ×100 mm column, Waters, Massachusetts, United States). The temperature of the column was set at 45°C and the injection volume was 3 μL. The mobile phase consisted of phases A and B. Mobile phase A was constituted using ammonium acetonitrile formate aqueous solution (acetonitrile: water = 6:4, v/v) and mobile phase B was ammonium formate acetonitrile isopropanol solution (acetonitrile: isopropanol = 1:9, v/v).

The samples were separated by UHPLC and analyzed on the Q Exactive Plus mass spectrometer (Thermo Scientific, Massachusetts, United States). The electrospray ionization (ESI) source conditions were as follows in the positive mode: heater temperature = 300°C; sheath gas flow rate = 45 arb; auxiliary gas flow rate = 15 arb; sweep gas flow rate = 1 arb; spray voltage = 3.0 kV; capillary temperature = 350°C; S-lens radio frequency (RF) level = 50%; and MS^1^ scan ranges = 200–1,800. In the negative mode: heater temperature = 300°C; sheath gas flow rate = 45 arb; auxiliary gas flow rate = 15 arb; sweep gas flow rate = 1 arb; spray voltage = 2.5 kV; capillary temperature = 350°C; S-Lens RF level = 60%; and MS^1^ scan ranges = 250–1,800. The mass charge ratio of lipid molecules and lipid fragments was determined according to the following protocol: 10 fragments (MS^2^ scan, high-energy collisional dissociation) were collected after each full scan. The resolution of MS^1^ at m/z 200 was 70,000, and that of MS^2^ was 17,500.

### Statistical Analysis

The raw MS data for the metabolomics were converted to MzXML files using ProteoWizard MS Convert and processed using XCMS for feature detection, retention time correction, and alignment. The metabolites were identified by accuracy mass (<25 ppm) and MS/MS data that were matched with our standards database. LipidSearch software version 4.1 (Thermo Scientific™) was used for feature detection, retention time correction, and alignment.

In the extracted ion features, only variables having more than 50% of the non-zero measurement values in at least one group were retained. The data were normalized by the total peak area. SIMCA-P14.1 (Umetrics, Umea, Sweden) was used for pattern recognition multivariate statistical analysis. After Pareto scaling, principal component analysis (PCA), partial least squares discriminant analysis (PLS-DA), and orthogonal partial least squares discriminant analysis (OPLS-DA) were performed. PCA was used to visualize the dataset, including the similarities and differences. The PLS-DA model was validated by 7-fold permutation tests. OPLS-DA was performed to maximize the covariance between the measured data and the response variable. To avoid over-fitting, model validity was evaluated by a permutation test (14). Significantly different metabolites were metabolites with variable influence on projection (VIP) values from the OPLS-DA model >1.0 and *p-*values <0.05 for two-tailed Student's *t*-tests of the raw data.

Differential metabolites were further identified using the Kyoto Encyclopedia of Genes and Genomes (KEGG) online database (http://www.kegg.jp/). The KEGG pathway enrichment of the differentially expressed metabolites was assessed using Fisher's Exact Test; *P* < 0.05 was the significance threshold. The volcano map was created using R software (Version 3.5.1, R Foundation for Statistical Computing, Vienna, Austria).

Differences in milk composition, biochemical indices, hormones, and immune parameters were analyzed by *t*-test (SPSS version 18, IBM, New York, NY, United States). Milk composition data, biochemical parameters, hormone levels, and immune indices were the test variables, and the EUL and CTR were used as grouping variables. Differences were reported as significant at *P* < 0.05; *P* < 0.01 was considered highly significant and 0.05 ≤ *P* < 0.10 was designated as a tendency toward significance.

Simple correlations between differential metabolites and inflammatory factors (IL-6 and IL-8) were analyzed with Pearson correlation analysis, and multiple linear regressions among differential metabolites and inflammatory factors (IL-6 and IL-8) were analyzed by stepwise correlation analysis.

## Results

### Effects of EUL on General Milk Composition

General milk composition in the CTR and EUL groups are presented in Table 2. These data demonstrate that total milk solids exhibited a trend toward increasing (*P* = 0.093) in the EUL group. There were no significant differences in general milk fat, protein, or lactose between groups (*P* > 0.10).

**Table 2:** Effects of EUL on dry matter intake and milk yield and general composition (*n* = 10).

**Item**	**CTR**	**EUL**	**SEM**	***P***
Dry matter intake (kg/d)	19.81	19.62	0.91	0.562
Milk yield (kg/d)	23.95	24.64	1.42	0.635
Milk fat (%)	6.07	5.59	0.284	0.116
Milk protein (%)	3.34	3.44	0.068	0.176
Milk lactose (%)	4.83	4.79	0.076	0.612
Total milk solids (%)	14.60	13.93	0.357	0.093
Somatic cell count, SCC (10^5^/mL)	21.67	14.62	3.92	0.102

*CTR, basal diet; EUL, basal diet + 3% Eucommia ulmoides leaves; SEM, Standard error of mean*.

### Effects of EUL on Biochemical Indicators, Hormone Concentrations, and Immune Parameters in Milk

Though the general composition remained stable, the specific composition and properties, such as antioxidant parameters, immune indicators, and relevant hormones, showed a large difference between groups (Table 3). These data demonstrate that the EUL group exhibited a trend toward increased SOD levels (*P* = 0.059). Further, INS was significantly increased in the EUL group compared with the CTR group (*P* < 0.05), whereas there were no significant differences in other hormone indices. Levels of the immune indicators, IL-6 and IL-8, were substantially reduced (*P* < 0.05), whereas the CD4/CD8 ratio was markedly increased (*P* < 0.05) in the EUL group relative to the CTR group; other immune indicators did not exhibit significant differences.

**Table 3:** Biochemical indicators, hormone concentrations, and immune parameters in milk (*n* = 10).

**Items**	**CTR**	**EUL**	**SEM**	***P***
**Antioxidant parameters**				
SOD (μg/mL)	1.14	1.37	0.105	0.059
GSH-Px (ng/mL)	15.38	16.51	0.964	0.271
GR (ng/mL)	1.97	1.48	0.284	0.112
MDA (ng/mL)	5.56	5.24	0.312	0.327
**Hormone concentrations**				
GH (ng/mL)	4.76	5.15	0.858	0.662
T3 (ng/mL)	168.05	185.60	15.74	0.291
INS (ng/mL)	0.63	0.80	0.067	0.028
GC (ng/mL)	2.56	2.61	0.285	0.86
CORT (ng/mL)	102.69	95.34	7.61	0.357
ADR (ng/mL)	8.25	8.37	0.886	0.896
**Immune indicators**				
IgA (mg/mL)	0.98	0.94	0.068	0.619
IgG (mg/mL)	3.72	4.20	0.448	0.309
TNF-α (pg/mL)	88.61	86.14	6.69	0.72
IL-1β (pg/mL)	73.93	58.57	12.13	0.234
IL-6 (pg/mL)	191.31	105.43	20.76	0.008
IL-8 (pg/mL)	68.56	56.32	3.16	0.003
IFN-γ (pg/mL)	55.54	63.78	5.98	0.198
CD4 (ng/mL)	3.97	4.24	0.461	0.573
CD8 (ng/mL)	1.99	1.63	0.197	0.102
CD4/CD8	2.00	2.62	0.174	0.005

*CTR, basal diet; EUL, basal diet + 3% Eucommia ulmoides leaves; SEM, standard error of mean; SOD, superoxide dismutase; GSH-Px, glutathione peroxidase; GR, glutathione reductase; MDA, malondialdehyde; GH, growth hormone; T3, triiodothyronine; INS, insulin; GC, glucagon; CORT, cortisol; ADR, adrenaline; IgA, immunoglobulin A; IgG, immunoglobulin G; TNF-α, tumor necrosis factor-α; IL-1β, interleukin-1β; IL-6, interleukin-6; IL-8, interleukin-8; IFN-γ, interferon-γ; CD4, cluster of differentiation 4; CD8, cluster of differentiation 8*.

### Metabolomics

#### Analysis of Differential Metabolites in the CTR and EUL Groups

**Figure 1:**
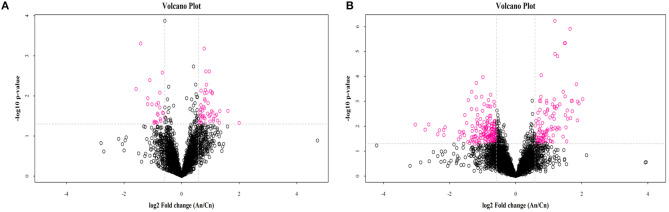
Volcano plot of metabolomics showing differential substances between the EUL and CTR groups. Each dot represents one compound. The x-axis represents log2 (Fold change), and the y-axis represents −log10 (*P-*value). The two lines parallel to the y-axis are x = −0.585 and x = 0.585. The points to the left of x = −0.585 and to the right of x = 0.585 are compounds with differences >1.5-fold. The line parallel to the x-axis are y = 1.30. Dots above the broken line indicate significant difference. Red points represent metabolites that differed significantly between the EUL and CTR groups, according the criteria, fold change >1.5 and *t*-test *P* < 0.05 in positive **(A)** and negative **(B)** modes. An = EUL, Basal diet + 3% *Eucommia ulmoides* leaves; Cn = CTR, Basal diet.

OPLS-DA results for the two groups showed clear separation and discrimination between the CTR and EUL groups, indicating that there was a notable difference between them (Supplementary Figure 1). Figure 1 shows different metabolites according to FC > 1.5 and *P* < 0.05 in positive and negative mode between two groups. Metabolites meeting the criteria, a variable importance in projection (VIP) value > 1, and *P* < 0.05 indicated a significant difference between the groups (Table 4). Twenty-five differential metabolites were identified between the CTR and EUL groups; 14, including 7Z, 10Z, 13Z, 16Z, 19Z-docosapentaenoic acid (FC = 3.129), adrenic acid (FC = 2.830), and eicosapentaenoic acid (FC = 1.685), were significantly increased in the EUL group whereas 11 metabolites, including indole-2-carboxylic acid (FC = 0.636), cholic acid (FC = 0.430), and creatine (FC = 0.784), were significantly decreased (*P* < 0.05).

**Table 4:** Differential metabolites in milk between the EUL and CTR groups (*n* = 10, CTR and *n* = 8, EUL).

**Description**	**VIP**	**FC**	**FDR**	**m/z**	**Rt (s)**
Beta-citronellol	2.325	0.732	0.021	198.18	53.21
L-carnitine	1.967	1.774	0.022	144.10	659.24
L-palmitoylcarnitine	2.082	2.086	0.022	400.34	362.44
5-aminopentanoic acid	1.224	1.327	0.039	118.09	674.23
Indole-2-carboxylic acid	2.310	0.636	0.039	162.05	57.82
PC (16:0/16:0)	1.887	1.558	0.039	756.55	309.97
Levonordefrin	2.240	0.559	0.039	206.08	360.44
1,2-dioleoyl-sn-glycero-3-hosphatidylcholine	1.808	1.701	0.039	808.58	301.65
1-stearoyl-2-oleoyl-sn-glycero-3-phosphoethanolamine	1.909	1.561	0.039	790.53	306.81
D-lactose	1.526	1.353	0.047	325.11	798.38
Guanosine	1.656	0.555	0.048	266.09	269.68
7Z, 10Z, 13Z, 16Z, 19Z-docosapentaenoic acid	6.411	3.129	0.000	329.25	96.52
Adrenic acid	4.352	2.830	0.000	331.26	95.53
Arachidonic acid (peroxide free)	8.231	2.281	0.000	303.23	97.21
Norethindrone acetate	7.347	3.272	0.005	339.20	60.62
Eicosapentaenoic acid	2.708	1.685	0.005	301.22	98.31
16-hydroxypalmitic acid	4.191	0.694	0.021	271.23	111.10
Alpha-D-glucose	4.180	0.602	0.025	179.06	538.84
Cholic acid	1.243	0.430	0.039	407.28	399.55
Benzoic acid	4.498	2.705	0.039	121.03	208.55
1-palmitoyl-2-hydroxy-sn-glycero-3-phosphoethanolamine	1.392	1.516	0.039	452.28	363.88
Dihydroxyacetone	2.713	0.742	0.039	111.01	840.83
Creatine	1.235	0.784	0.039	130.06	629.63
Cytidine monophosphate N-acetylneuraminic acid	2.039	0.439	0.047	613.14	796.84
N-acetylmannosamine	4.745	0.603	0.047	220.08	486.82

*CTR, basal diet; EUL, basal diet + 3% Eucommia ulmoides leaves; VIP, variable importance in projection; FC, fold-change, mean value of peak area obtained from the EUL group/mean value of peak area obtained from the CTR group. FC values>1 indicate that metabolites are present at higher levels in the EUL group than the CTR group and FC values <1 indicate that metabolites are present in the EUL group less than the CTR group. m/z, mass-to-charge ratio; Rt, retention time*.

#### Pathways Analysis of Differential Metabolites

**Figure 2:**
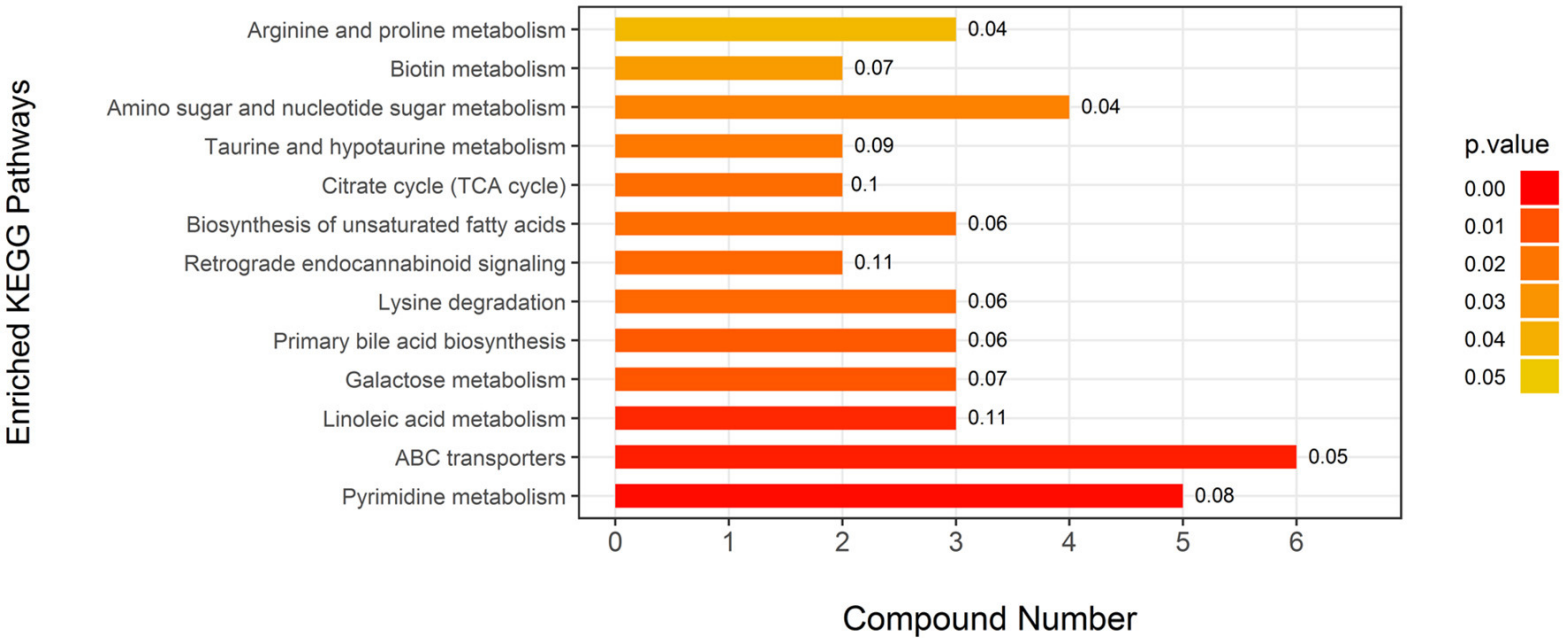
Key differential metabolic pathways between the EUL and CTR groups. This figure aims to find pathways significant changed based on enrichment and topology analysis. The x-axis represents compound numbers, and the y-axis represents enriched KEGG pathways. The colors represent pathway impact values. The numbers to the right of the bars in the graph mean the proportion of differential metabolites in the total number of metabolites in this pathway.

Attributive analysis using KEGG identified 25 differential metabolites as enriched in 13 metabolic pathways (Figure 2) involved in amino acid metabolism, carbohydrates metabolism, fatty acid metabolism, and energy metabolism, indicating that EUL influences many biochemical metabolic pathways to affect milk quality. Visually interpreting a metabolome map, linoleic acid metabolism, and retrograde endocannabinoid signaling were the pathways most impacted by EUL (Figure 2). The ABC transporters are one of the most impacted pathways by EUL.

### Lipidomics

**Figure 3:**
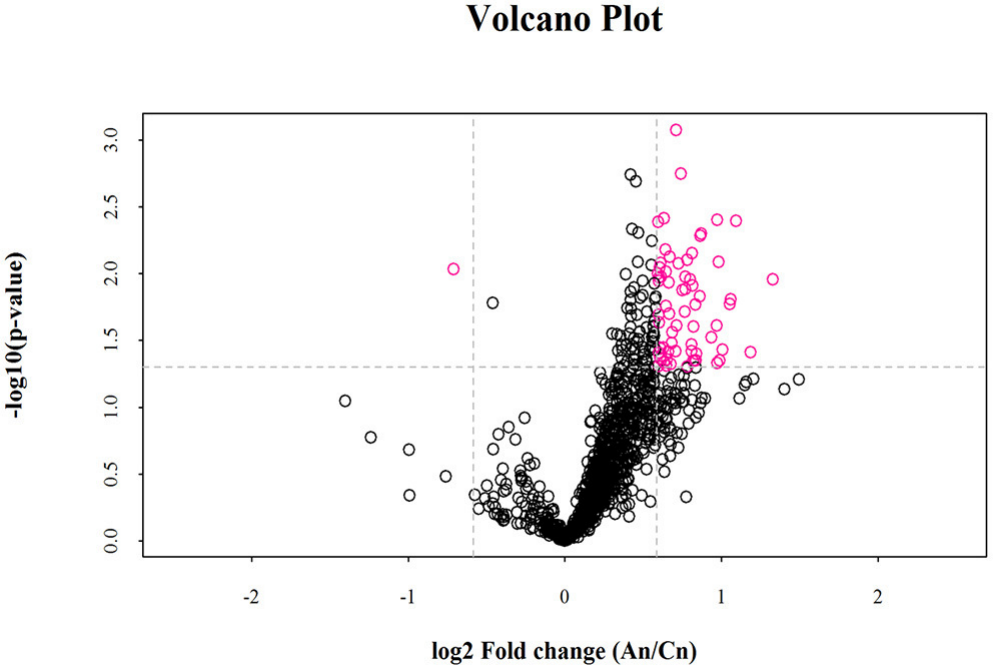
Volcano plot of lipidomics illustrating compounds differing between the EUL and CTR groups. Each dot represents one compound. The x-axis represents log2 (Fold change), and the y-axis represents –log10 (*P-*value). The two lines parallel to the y-axis are x = −0.585 and x = 0.585. The points to the left of x = −0.585 and to the right of x = 0.585 are compounds with differences >1.5-fold. The line parallel to the x-axis are y = 1.30. Dots above the broken line indicate significant difference. Red points represent metabolites differing significantly, based on fold-change > 1.5 and *P* < 0.05 (*t*-test). An = EUL, Basal diet + 3% *Eucommia ulmoides* leaves; Cn = CTR, Basal diet.

**Figure 4:**
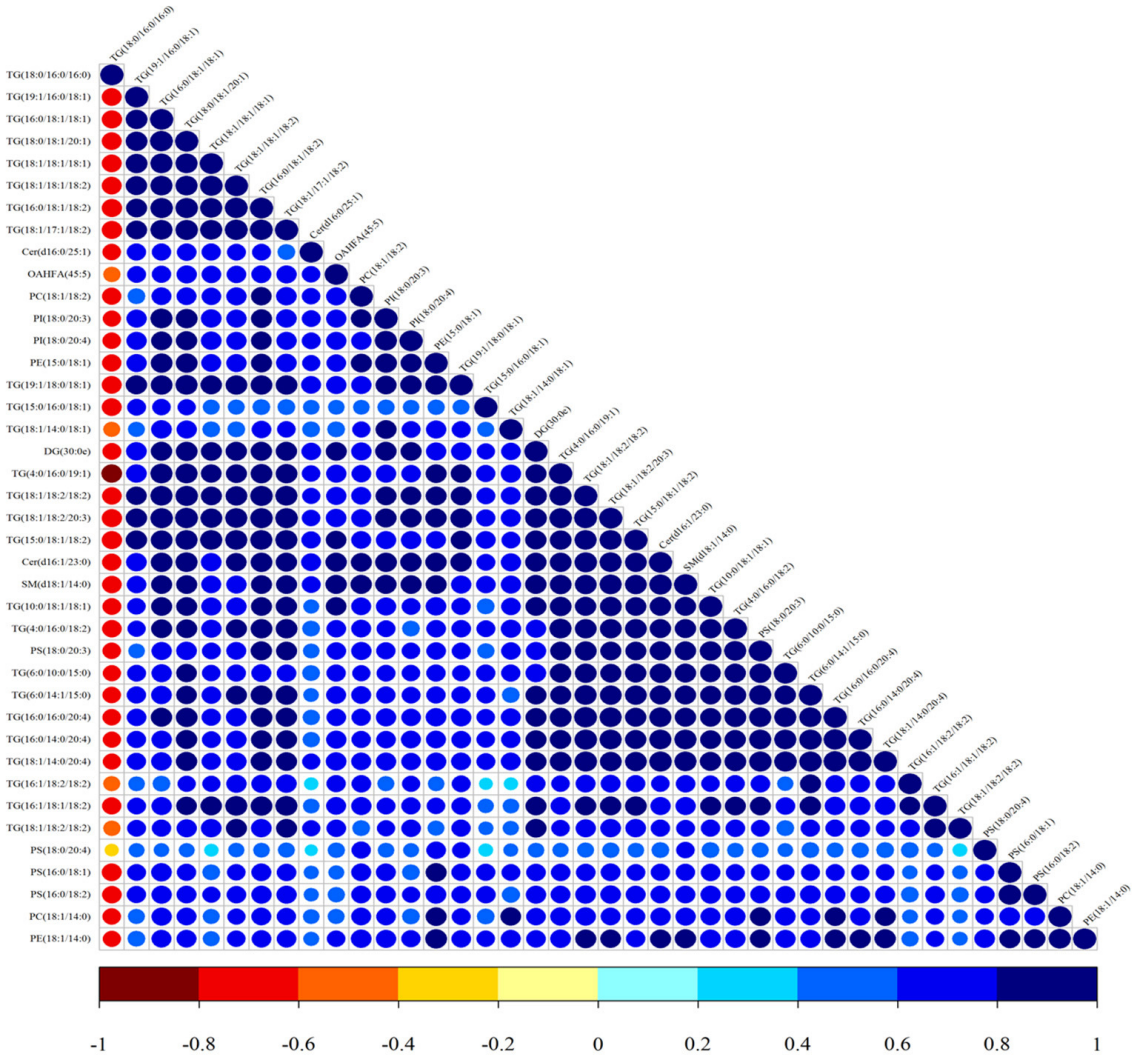
Correlation analysis of lipid molecules in the CTR and EUL groups. Map illustrating the correlations among lipid species differing significantly between EUL and CTR groups. The color of the points represents the degree of correlation, varying from −1 to 1, from jujube red to blue, respectively.

Lipid composition of the milk, determined by lipidomic analysis, is presented in Supplementary Figure 2. A total of 21 lipid classes, including 1,094 lipid species, were detected in the milk. The results of OPLS-DA analysis of lipid metabolites are presented in Supplementary Figure 3. The data show that lipid metabolite levels were clearly separated in the different experimental groups, indicating that EUL has a substantial effect on lipid metabolism in dairy cows. Further analysis of the differential compounds was conducted by generating volcano plots (Figure 3). The metabolites screened (VIP > 1 and *P* < 0.05) are listed in Table 5. Among lipid species, 40 differed significantly between the two groups, including those in the triglyceride (TG), phosphatidyl choline (PC), phosphatidyl ethanolamine (PE), phosphatidyl serine (PS), phosphatidyl inositol (PI), sphingomyelin (SM), ceramide (Cer), (O-acyl) omega-hydroxy fatty acids, and diglyceride categories (Table 5). Of those 40 lipid species, 39 were significantly higher in the EUL, with only one present at lower levels in the EUL (TG; 18:0/16:0). Correlation analysis of lipid molecules that differed significantly between the groups (Figure 4) indicated that TG (18:0/18:0/16:0)+NH_4_ was negatively correlated with the other 39 substances, while the negative correlation with TG (4:0/16:0/19:1)+NH_4_ was strongest (*R*^2^ = −0.81).

**Table 5:** Differences in lipid metabolites content between the CTR and EUL groups (*n* = 10, CTR and *n* = 8, EUL).

**Lipid ion**	**Class**	**Ion formula**	**Cal Mz**	**RT-(min)**	**VIP**	**FC**	**FDR**
TG(6:0/10:0/15:0)+NH_4_	TG	C34 H68 O6 N1	586.50	13.32	1.15	1.525	0.046
TG(6:0/14:1/15:0)+NH_4_	TG	C38 H74 O6 N1	640.55	14.64	1.94	1.630	0.046
OAHFA(45:5)-H	OAHFA	C45 H77 O4	681.58	15.18	1.15	2.071	0.038
TG(4:0/16:0/18:2)+NH_4_	TG	C41 H78 O6 N1	680.58	15.55	1.83	1.357	0.038
TG(4:0/16:0/19:1)+NH_4_	TG	C42 H82 O6 N1	696.61	17.08	2.64	1.494	0.039
PC(18:1/14:0)+HCOO	PC	C41 H79 O10 N1 P1	776.54	11.35	1.30	1.290	0.046
PC(18:1/18:2)+HCOO	PC	C45 H83 O10 N1 P1	828.58	11.66	2.59	1.328	0.045
TG(10:0/18:1/18:1)+NH_4_	TG	C49 H94 O6 N1	792.71	20.49	4.96	1.410	0.039
PE(18:1/14:0)-H	PE	C37 H71 O8 N1 P1	688.49	11.64	1.05	1.316	0.046
PE(15:0/18:1)-H	PE	C38 H73 O8 N1 P1	702.51	12.31	1.03	1.360	0.046
TG(15:0/16:0/18:1)+NH_4_	TG	C52 H102 O6 N1	836.77	22.23	1.40	1.820	0.038
TG(18:0/16:0/16:0)+NH_4_	TG	C53 H106 O6 N1	852.80	24.08	2.48	0.726	0.038
DG(30:0e)+Na	DG	C33 H66 O4 Na1	549.49	22.62	1.09	1.336	0.036
TG(18:1/14:0/18:1)+NH_4_	TG	C53 H102 O6 N1	848.77	22.72	3.47	1.257	0.045
TG(16:0/14:0/20:4)+NH_4_	TG	C53 H98 O6 N1	844.74	21.40	1.19	1.569	0.046
TG(15:0/18:1/18:2)+NH_4_	TG	C54 H102 O6 N1	860.77	22.31	2.83	2.131	0.039
TG(16:0/18:1/18:1)+NH_4_	TG	C55 H106 O6 N1	876.80	23.45	4.00	1.231	0.045
TG(16:0/18:1/18:2)+NH_4_	TG	C55 H104 O6 N1	874.79	22.80	4.19	1.339	0.038
TG(16:1/18:1/18:2)+NH_4_	TG	C55 H102 O6 N1	872.77	22.01	2.82	1.529	0.039
TG(16:0/16:0/20:4)+NH_4_	TG	C55 H102 O6 N1	872.77	22.47	1.34	1.445	0.049
TG(16:1/18:2/18:2)+NH_4_	TG	C55 H100 O6 N1	870.75	21.06	1.01	1.752	0.046
TG(18:1/14:0/20:4)+NH_4_	TG	C55 H100 O6 N1	870.75	21.42	1.23	1.698	0.039
TG(19:1/16:0/18:1)+NH_4_	TG	C56 H108 O6 N1	890.82	23.78	2.61	1.586	0.039
TG(18:1/17:1/18:2)+NH_4_	TG	C56 H104 O6 N1	886.79	22.38	1.04	1.780	0.039
TG(18:1/18:1/18:1)+NH_4_	TG	C57 H108 O6 N1	902.82	23.50	3.08	1.277	0.045
TG(18:1/18:1/18:2)+NH_4_	TG	C57 H106 O6 N1	900.80	22.86	2.93	1.374	0.039
TG(18:1/18:2/18:2)+NH_4_	TG	C57 H104 O6 N1	898.79	22.03	2.17	1.716	0.039
TG(18:1/18:2/18:2)+NH_4_	TG	C57 H104 O6 N1	898.79	22.48	1.81	1.563	0.039
TG(19:1/18:0/18:1)+NH_4_	TG	C58 H112 O6 N1	918.85	24.40	1.76	1.957	0.045
TG(18:0/18:1/20:1)+NH_4_	TG	C59 H114 O6 N1	932.86	24.68	1.28	1.355	0.046
TG(18:1/18:2/20:3)+NH_4_	TG	C59 H106 O6 N1	924.80	22.49	1.52	1.703	0.039
Cer(d16:1/23:0)+HCOO	Cer	C40 H78 O5 N1	652.59	16.01	4.45	1.786	0.046
PI(18:0/20:3)-H	PI	C47 H84 O13 N0 P1	887.57	12.18	1.49	1.522	0.046
PI(18:0/20:4)-H	PI	C47 H82 O13 N0 P1	885.55	11.29	3.11	1.489	0.046
PS(16:0/18:1)-H	PS	C40 H75 O10 N1 P1	760.51	11.44	2.37	1.562	0.039
PS(16:0/18:2)-H	PS	C40 H73 O10 N1 P1	758.50	10.79	1.47	1.668	0.036
PS(18:0/20:3)-H	PS	C44 H79 O10 N1 P1	812.54	11.67	3.77	1.514	0.046
PS(18:0/20:4)-H	PS	C44 H77 O10 N1 P1	810.53	13.06	1.55	1.337	0.039
SM(d18:1/14:0)+HCOO	SM	C38 H76 O8 N2 P1	719.53	10.14	1.74	1.346	0.039
Cer(d16:0/25:1)+HCOO	Cer	C42 H82 O5 N1	680.62	17.89	1.23	1.983	0.046

*CTR, basal diet; EUL, basal diet + 3% Eucommia ulmoides leaves; TG, triglyceride; OAHFA, (O-acyl) omega-hydroxy fatty acids; PC, phosphatidyl choline; PE, phosphatidyl ethanolamine; DG, diglyceride; Cer, ceramide; PI, phosphatidyl inositol; PS, phosphatidyl serine; SM, sphingomyelin; VIP, variable importance in projection; FC, fold-change, FC values > 1 indicate that metabolites are present at higher levels in the EUL group than the CTR group and FC values <1 indicate that metabolites are present in the EUL group less than the CTR group. m/z, mass-to-charge ratio; Rt, retention time*.

### The Correlation Analysis Between Differential Metabolites and Inflammatory Factors

Pearson correlation analysis was used to assess the relationships between differential metabolites and inflammatory factors (IL-6 and IL-8) (Table 6). Metabolites and lipid species, such as 7Z, 10Z, 13Z, 16Z, 19Z-docosapentaenoic acid, adrenic acid, and eicosapentaenoic acid up-regulated in EUL, were negatively correlated with the inflammatory factors IL-6 and IL-8 (*P* < 0.05). Conversely, the metabolites 16-hydroxypalmitic acid and alpha-D-Glucose, which were significantly down-regulated in EUL, were positively correlated with IL-6 and IL-8 (*P* < 0.05). Further, multiple linear regression analysis was used to conduct stepwise correlations between differential metabolites selected based on Pearson correlation analysis and inflammatory factors (Table 7). There was a negative correlation between eicosapentaenoic acid and IL-6 (*P* < 0.05) whereas 7Z, 10Z, 13Z, 16Z, 19Z-docosapentaenoic acid, PC (16:0/16:0), and adrenic acid were negatively correlated with IL-8 (*P* < 0.05), while 16-hydroxypalmitic acid and IL-8 were positively correlated (*P* < 0.05). These findings indicate that EUL may improve milk quality by reducing the levels of inflammatory factors.

**Table 6:** Simple correlation analysis between differential metabolites and inflammatory factors.

**Inflammatory factors**	**Differential metabolites**	**Correlation coefficient**	***P***
IL-6	7Z, 10Z, 13Z, 16Z, 19Z-Docosapentaenoic acid	−0.693	0.013
	PC (16:0/16:0)	−0.698	0.012
	Adrenic Acid	−0.631	0.028
	Arachidonic Acid (peroxide free)	−0.734	0.007
	Eicosapentaenoic acid	−0.705	0.01
	16-Hydroxypalmitic acid	0.642	0.024
	Alpha-D-Glucose	0.733	0.007
	Cytidine monophosphate N-acetylneuraminic acid	0.687	0.014
	TG (4:0/16:0/19:1)+NH4	−0.669	0.017
	TG (10:0/18:1/18:1)+NH4	−0.58	0.048
	TG (15:0/16:0/18:1)+NH4	−0.577	0.05
	TG (18:0/16:0/16:0)+NH4	0.587	0.045
	SM (d18:1/14:0)+HCOO	−0.617	0.032
IL-8	7Z, 10Z, 13Z, 16Z, 19Z-Docosapentaenoic acid	−0.772	0.003
	PC (16:0/16:0)	−0.773	0.003
	Adrenic Acid	−0.745	0.005
	Norethindrone Acetate	−0.658	0.002
	Eicosapentaenoic acid	−0.627	0.03
	16-Hydroxypalmitic acid	0.807	0.002
	Alpha-D-Glucose	0.749	0.005

**Table 7:** Multiple linear regression analysis of the relationships among differential metabolites and inflammatory factors.

**Inflammatory factors**	**Differential metabolites**	**Correlation coefficient**	***P***
IL-6	Eicosapentaenoic acid	−0.705	0.01
IL-8	7Z, 10Z, 13Z, 16Z, 19Z-Docosapentaenoic acid	−0.772	0.003
	PC (16:0/16:0)	−0.773	0.003
	Adrenic Acid	−0.745	0.005
	16-Hydroxypalmitic acid	0.807	0.002

## Discussion

Milk composition can be affected by several factors such as genetics, the physiological and nutritional status of the animal, animal management, and environmental conditions (15, 16). EUL is a dual functional feed, which provides both nutrition and bioactive components such as geniposide acid, aucubin, and chlorogenic acid, which have specific functions in enhancing immunity. Chlorogenic acid can increase antioxidation capacity; for example, by improving SOD and GSH-Px activities and reducing oxidation products, such as MDA, in pigs, chickens, and sheep, thereby improving product quality (4, 5, 17). In China, EUL is routinely served as food, tea, medicine, and animal feed additives in rural communities (7, 18). Therefore, it may have an influence on the metabolism and chemical and physical properties of dairy cows.

In our study, IL-6 and IL-8 were lower in the EUL group than in the CTR group, indicating that the bioactive components in EUL, such as chlorogenic acid and aucubin, act as inhibitors of inflammatory factors as well as improving antioxidation, eliminating free radicals, and upregulating SOD activity (19, 20). Chlorogenic acid can significantly suppress mRNA expression of the proinflammatory molecules IL-6 and IL-8, thus alleviating inflammatory responses in bovine mammary epithelial cells (21, 22). The CD4/CD8 ratio is another indicator reflecting physiological immune status (23). In the present study, CD4/CD8 was significantly increased in the EUL group, indicating that EUL improves mammary gland immunity and milk quality.

Metabolites reflect alterations in the metabolism of dairy cows. Based on the current study, 25 differential metabolites were identified between the CTR and EUL groups including amino acids, carbohydrates, and lipids. Our data demonstrate that INS was significantly increased in the EUL group compared with the CTR group, whereas Alpha-D-glucose was significantly reduced. As the nutrients in milk are transported from the blood, variations in the blood also affect the composition of the milk. According to previous reports, EUL significantly reduced blood glucose levels and significantly increased INS levels (24). In addition, the milk transfer of compounds is mainly mediated by two transporter superfamilies: ATP-binding cassette and Solute Carrier (25). In our study, one of the most impacted pathways of EUL was the ABC transporters, which was mainly associated with changes in amino acids (Taurine, L-Lysine) and carbohydrates (D-Lactose) between the CTR and EUL groups. This may partly explain the variations in the levels of Alpha-D-glucose and INS between CTR and EUL groups.

Milk fat content and milk fatty acid composition are mainly affected by animal diet (26). In the current study, polyunsaturated fatty acids (PUFA), including 7Z, 10Z, 13Z, 16Z, 19Z-docosapentaenoic acid, adrenic acid, eicosapentaenoic acid, and arachidonic acid, were increased in the EUL group. Short-chain fatty acids and UFA are beneficial to human health (27). Eicosapentaenoic acid and 7Z, 10Z, 13Z, 16Z, 19Z-docosapentaenoic acid are n-3 PUFA molecules, which have benefits in preventing cardiovascular disease and in being anti-inflammatory (28), and were found to have significantly increased in the present study, indicating that the nutritional value of milk was improved in the EUL group. In previous studies, it was found that the amount of fatty acid in feed determines the fatty acid profile in dairy products (29). EUL groups are rich in linolenic acid, which is the precursor of 7Z, 10Z, 13Z, 16Z, 19Z-docosapentaenoic acid and eicosapentaenoic acid, which explains the increase of 7Z, 10Z, 13Z, 16Z, 19Z-docosapentaenoic acid and eicosapentaenoic acid in milk. In line with this, Zhou et al. also observed that chlorogenic acid increased C18:3n-3 content in the liver of rats (30). Adrenic acid is an essential n-6 unsaturated fatty acid associated with boosting immunity, brain and nervous system development, and prevention of cardiovascular disease and diabetes (31), and was enriched in the EUL group. This is related to linolenic acid and linoleic acid, the precursors of adrenic acid, which are abundant in the EUL group. The increase of PUFA in the EUL group is favorable because a high content of PUFA has become a desirable nutritional characteristic in livestock products for consumers (32). The most enriched pathway in our study was the linoleic acid metabolism signaling pathway, which was mainly associated with changes in PUFA between the EUL and CTR groups. The increasing content of PUFA in the EUL group was likely related to the following factors: (1) EUL are rich in PUFA, such as linoleic acid, α-linoleic acid, and adrenic acid, which can increase the levels of precursors (7); (2) active substances in EUL, such as lignin, iridoid terpenes, and flavonoids, can affect fat metabolism and SFA synthesis (2, 33).

Lipidomics can be used to quantify total lipid components (34). Milk fat is an important source of essential fatty acids and a carrier of the lipid soluble vitamins, A, D, and E (35). The main component of milk fat is triglyceride (TG), which accounts for ~95% of total milk fat. Compared with the CTR group, 40 differential metabolites were identified in the EUL group by lipidomics analysis. EUL had a significant effect on TG composition in milk. Twenty-three types of TG with polyunsaturated bonds were increased and TG (18:0/16:0/16:0)+NH_4_ decreased; however, total TG content was not significantly altered. This is because chlorogenic acid supplementation reduced the SFA content, which is consistent with previous studies on broiler chicken meat (5). Furthermore, TG (18:0/16:0/16:0)+NH_4_ was negatively correlated with the 39 other differential lipids. It was reported that consumption of TG types with more carbon atoms and unsaturated bonds are associated with a lower incidence rate of type 2 diabetes (36). Hence, EUL substantially influenced milk lipid composition and may benefit the health of milk consumers.

Simple correlation and multiple linear regression analyses of differential metabolites with IL-6 and IL-8 revealed significant negative correlations of eicosapentaenoic acid, PC (16:0/16:0), and adrenic acid with IL-6 and IL-8, whereas 16-hydroxypalmitic acid was significantly positively correlated with IL-6 and IL-8. Some studies have reported that PUFA plays a key role in modulation of the immune system and inflammatory responses (37). Rezamand et al. reported that α-linolenic acid-enriched diets down-regulated expression of IL-6 and IL-8 pro-inflammatory factors (38). In our study, we found that eicosapentaenoic acid and adrenic acid significantly negatively correlated with inflammatory factors, but 16-hydroxypalmitic significantly positively correlated with inflammatory factors. Therefore, eicosapentaenoic acid, PC (16:0/16:0), and adrenic acid may serve as potential biomarkers for better milk micro-composition and quality. Overall, our data suggest that the ingredients of EUL in the diet of dairy cows could endow milk with some desirable traits and benefit consumer health. However, the weak point of this work is that before the EUL treatment, the only baseline data researched was on general milk composition, which did not include data from metabolomics and lipidomics analyses. In the future, we will carry out a time-course study to further clarify the associated underlying mechanisms of EUL in regulating milk quality.

## Conclusions

In summary, these results suggest that EUL reduced levels of proinflammatory factors IL-6 and IL-8 and increased the ratio of inflammatory inhibitory factors CD4/CD8. EUL had positive effects on unsaturated fatty acid and micro-component contents, which can improve the nutritional value of milk. The underlying mechanisms are related to the rich, natural, bioactive ingredients that serve multiple functions in EUL.

## Data Availability Statement

The original contributions presented in the study are included in the article/Supplementary Material, further inquiries can be directed to the corresponding author/s.

## Ethics Statement

All procedures were approved by the Animal Care and Use Committee of Henan Agricultural University, Henan Agricultural University, Zheng Zhou, China (approval number: HENAU-2018-015).

## Author Contributions

ZT: conceptualization, data curation, formal analysis, methodology, and writing-original draft. LW: project administration and validation. HD: funding acquisition and supervision. GY, TF, HL, and YS: formal analysis and visualization. SL and LZ: formal analysis, visualization, and validation. TG: project administration, funding acquisition, and supervision. All authors contributed to the article and approved the submitted version.

## Conflict of Interest

The authors declare that the research was conducted in the absence of any commercial or financial relationships that could be construed as a potential conflict of interest.

## Supplementary Material

Supplementary Figure 1 Positive and negative mode OPLS-DA score maps comparing EUL and CTR groups. **(A,C)** score maps in positive and negative mode, respectively. **(B,D)** corresponding OPLS-DA validation plots in positive mode and negative mode, respectively. Green, EUL group; blue, CTR group. Cn = CTR, diet without EUL; An = EUL, diet containing 3% EUL, dry matter basis.

Supplementary Figure 2 Lipid classes and lipid species differing significantly between the EUL and CTR groups.

Supplementary Figure 3 OPLS-DA score map between the EUL and CTR groups. **(A)** Score maps in positive and negative modes. **(B)** Corresponding validation plots of OPLS-DA in positive mode and negative mode. Blue, EUL group. Green, CTR group. CTR, Basal diet, diet without EUL; EUL, diet containing 3% EUL, dry matter basis.

Supplementary Table 1 Milk baseline data before the EUL treatment. CTR, basal diet; EUL, basal diet + 3% *Eucommia ulmoides* leaves; SEM, Standard error of mean.

Supplementary Table 2 Biochemical indicators, related hormones, and immune parameters in blood. CTR, basal diet; EUL (1%), basal diet + 1% *Eucommia ulmoides* leaves; EUL (3%), basal diet + 3% *Eucommia ulmoides* leaves; SEM, standard error of mean; SOD, superoxide dismutase; GSH-Px, glutathione peroxidase; GR, glutathione reductase; MDA, malondialdehyde; GH, growth hormone; T3, triiodothyronine; INS, insulin; GC, glucagon; CORT, cortisol; ADR, adrenaline; IgA, immunoglobulin A; IgG, immunoglobulin G; TNF-α, tumor necrosis factor-α; IL-1β, interleukin-1β; IL-6, interleukin-6; IL-8, interleukin-8; IFN-γ, interferon-γ; CD4, cluster of differentiation 4; CD8, cluster of differentiation 8.
