# Supplementary figures and images for: Metabolomic and Lipidomic Approaches to Evaluate the Effects of Eucommia ulmoides Leaves on Milk Quality and Biochemical Properties
Source: Front Vet Sci. 2021 Jun 1;8:644967. doi: 10.3389/fvets.2021.644967 (PMC8204049; doi:10.3389/fvets.2021.644967)

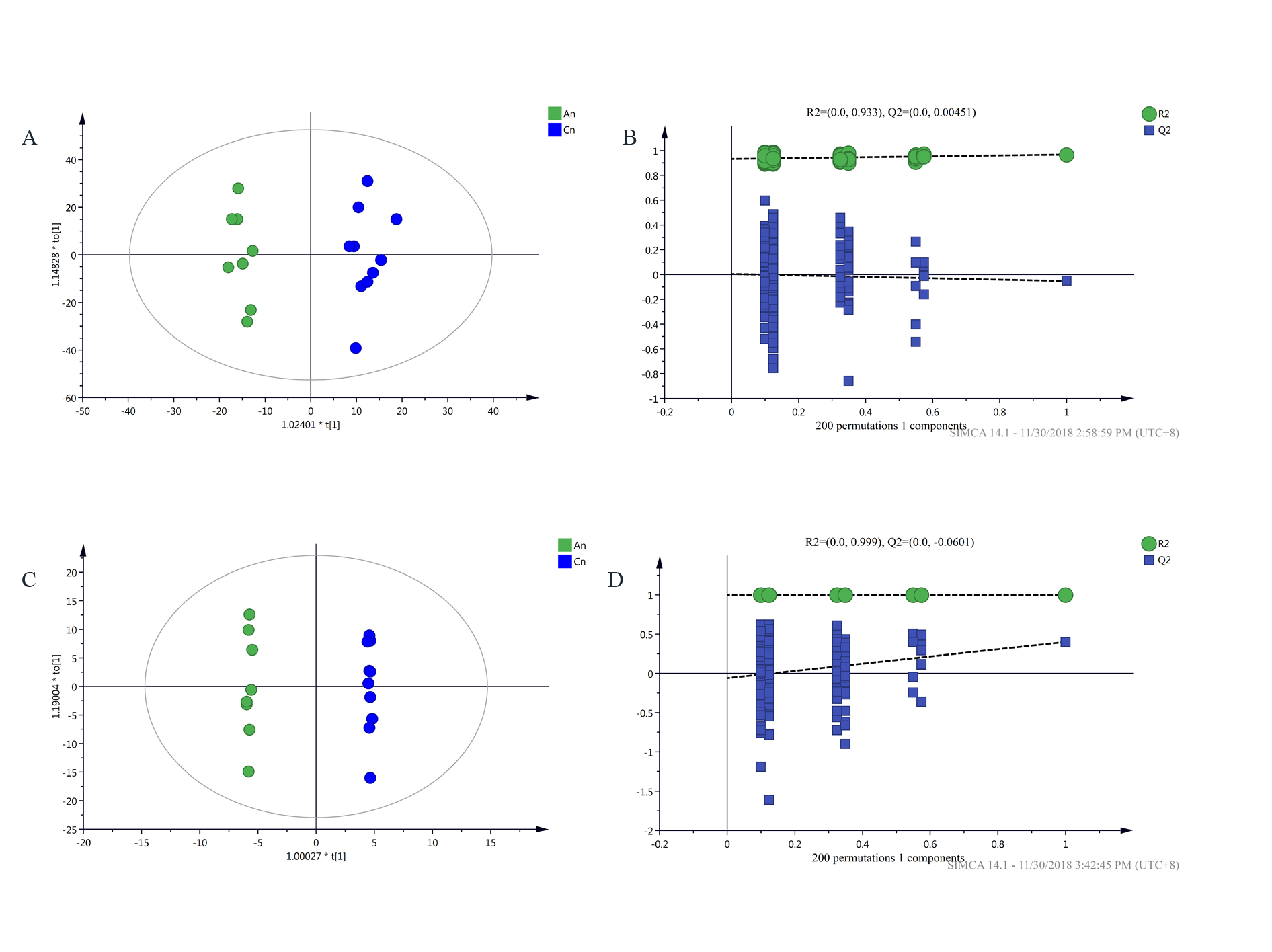

Supplement: Supplementary Figure 1 — Positive and negative mode OPLS-DA score maps comparing EUL and CTR groups. (A,C) score maps in positive and negative mode, respectively. (B,D) corresponding OPLS-DA validation plots in positive mode and negative mode, respectively. Green, EUL group; blue, CTR group. Cn = CTR, diet without EUL; An = EUL, diet containing 3% EUL, dry matter basis. [file Image_1.tif]

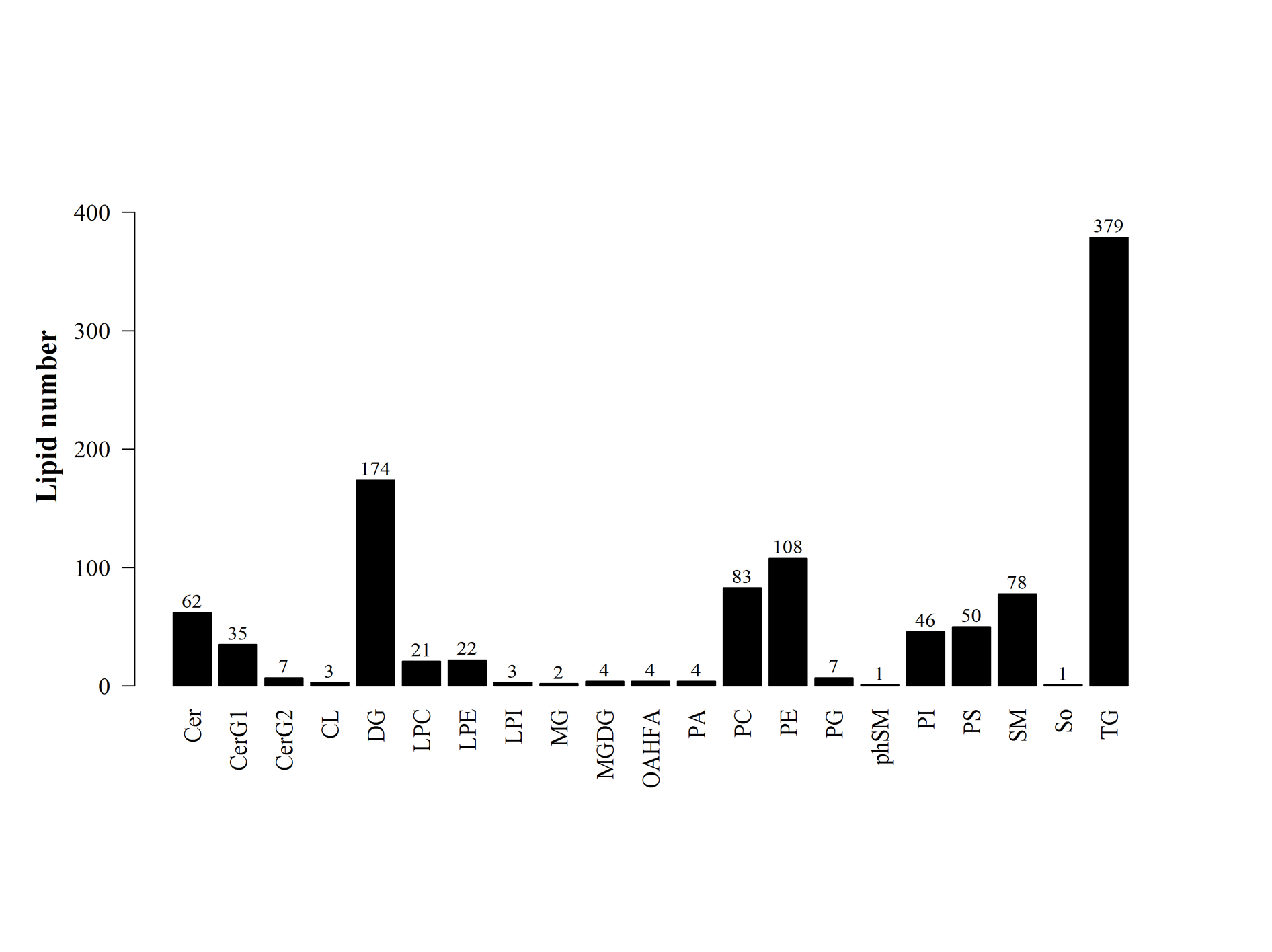

Supplement: Supplementary Figure 2 — Lipid classes and lipid species differing significantly between the EUL and CTR groups. [file Image_2.tif]

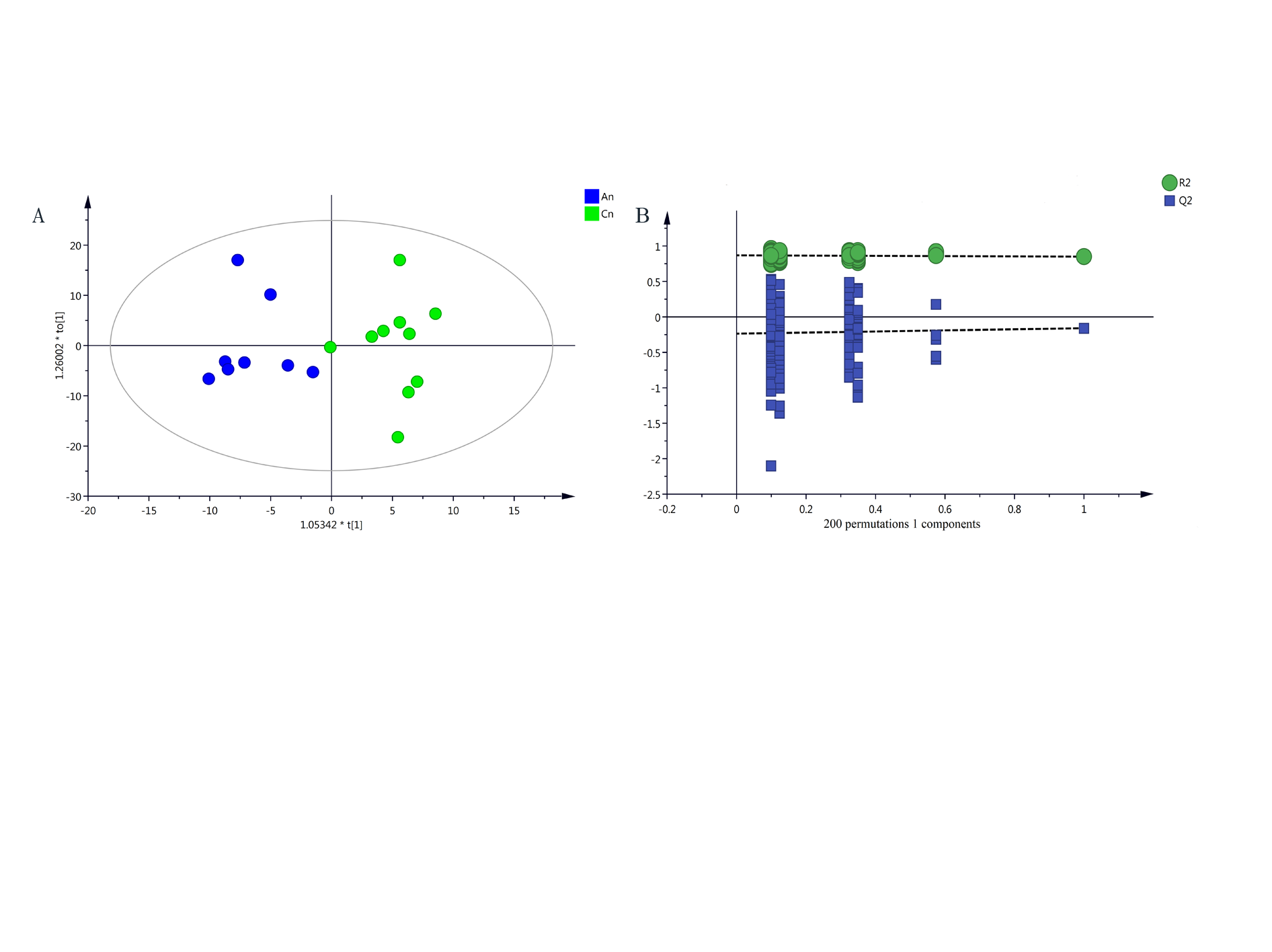

Supplement: Supplementary Figure 3 — OPLS-DA score map between the EUL and CTR groups. (A) Score maps in positive and negative modes. (B) Corresponding validation plots of OPLS-DA in positive mode and negative mode. Blue, EUL group. Green, CTR group. CTR, Basal diet, diet without EUL; EUL, diet containing 3% EUL, dry matter basis. [file Image_3.tif]
