# Supplementary material for: Metabolomic and Lipidomic Approaches to Evaluate the Effects of Eucommia ulmoides Leaves on Milk Quality and Biochemical Properties
Source: Front Vet Sci. 2021 Jun 1;8:644967. doi: 10.3389/fvets.2021.644967 (PMC8204049; doi:10.3389/fvets.2021.644967)
Supplement: Supplementary Table 1 — Milk baseline data before the EUL treatment. CTR, basal diet; EUL, basal diet + 3% Eucommia ulmoides leaves; SEM, Standard error of mean. [file Table_1.docx]

Table S1 Milk baseline data before the EUL treatment (n=10)

| Item | CTR | EUL | SEM | *P* |
| --- | --- | --- | --- | --- |
| Milk fat (%) | 6.41 | 5.60 | 0.65 | 0.231 |
| Milk protein (%) | 3.38 | 3.32 | 0.09 | 0.540 |
| Milk lactose (%) | 4.81 | 4.80 | 0.15 | 0.932 |
| Total milk solids (%) | 14.91 | 14.08 | 0.68 | 0.244 |
| Somatic cell count, SCC (10^5^/mL) | 24.94 | 23.99 | 9.70 | 0.923 |

Abbreviations: CTR, Basal diet; EUL, Basal diet + 3% *Eucommia ulmoides* leaves; SEM, Standard error of mean.
