# Supplementary material for: Metabolomic and Lipidomic Approaches to Evaluate the Effects of Eucommia ulmoides Leaves on Milk Quality and Biochemical Properties
Source: Front Vet Sci. 2021 Jun 1;8:644967. doi: 10.3389/fvets.2021.644967 (PMC8204049; doi:10.3389/fvets.2021.644967)
Supplement: Supplementary Table 2 — Biochemical indicators, related hormones, and immune parameters in blood. CTR, basal diet; EUL (1%), basal diet + 1% Eucommia ulmoides leaves; EUL (3%), basal diet + 3% Eucommia ulmoides leaves; SEM, standard error of mean; SOD, superoxide dismutase; GSH-Px, glutathione peroxidase; GR, glutathione reductase; MDA, malondialdehyde; GH, growth hormone; T3, triiodothyronine; INS, insulin; GC, glucagon; CORT, cortisol; ADR, adrenaline; IgA, immunoglobulin A; IgG, immunoglobulin G; TNF-α, tumor necrosis factor-α; IL-1β, interleukin-1β; IL-6, interleukin-6; IL-8, interleukin-8; IFN-γ, interferon-γ; CD4, cluster of differentiation 4; CD8, cluster of differentiation 8. [file Table_2.docx]

| Items | CTR | EUL (1%) | EUL (3%) | SEM | *P* |
| --- | --- | --- | --- | --- | --- |
| **Antioxidant parameters** | | | | | |
| SOD (µg/mL) | 2.88^b^ | 3.31^b^ | 3.66^a^ | 0.19 | 0.003 |
| GSH-Px (ng/mL) | 14.56 | 22.58 | 22.16 | 4.06 | 0.127 |
| GR (ng/mL) | 2.68 | 2.67 | 2.81 | 0.10 | 0.305 |
| MDA (ng/mL) | 5.55 | 5.39 | 4.91 | 0.97 | 0.789 |
| **Hormone concentrations** | | | | | |
| GH (ng/mL) | 4.67 | 4.59 | 5.16 | 0.26 | 0.096 |
| T3 (ng/mL) | 199.58 | 277.68 | 245.71 | 37.49 | 0.154 |
| INS (ng/mL) | 0.89^b^ | 1.12^a^ | 1.07^a^ | 0.10 | 0.079 |
| GC (ng/mL) | 2.69 | 2.53 | 2.70 | 0.08 | 0.196 |
| CORT (ng/mL) | 141.19 | 184.53 | 147.01 | 30.78 | 0.344 |
| ADR (ng/mL) | 7.72 | 9.04 | 7.43 | 0.86 | 0.183 |
| **Immune indicators** | | | | | |
| IgA (mg/mL) | 2.12^b^ | 2.57^a^ | 2.81^a^ | 0.17 | 0.005 |
| IgG (mg/mL) | 5.05^b^ | 6.62^a^ | 6.34^a^ | 0.47 | 0.012 |
| TNF-α (pg/mL) | 160.46 | 176.38 | 161.89 | 13.18 | 0.435 |
| IL-1β (pg/mL) | 206.07 | 238.63 | 227.51 | 29.52 | 0.550 |
| IL-6 (pg/mL) | 176.66 | 174.37 | 147.85 | 21.78 | 0.370 |
| IL-8 (pg/mL) | 171.18 | 176.46 | 173.71 | 22.22 | 0.972 |
| IFN-γ (pg/mL) | 60.11 | 81.87 | 81.60 | 12.96 | 0.198 |
| CD4 (ng/mL) | 3.84^b^ | 5.13^a^ | 5.20^a^ | 0.59 | 0.068 |
| CD8 (ng/mL) | 5.27 | 6.58 | 6.04 | 0.83 | 0.318 |
| CD4/CD8 | 0.73 | 0.79 | 0.87 | 0.07 | 0.157 |

**Table S2.** Biochemical indicators, hormone concentrations, and immune parameters in blood (n=10)

Abbreviations: CTR, Basal diet; EUL (1%), Basal diet + 1% *Eucommia ulmoides* leaves; EUL (3%), Basal diet + 3% *Eucommia ulmoides* leaves; SEM, standard error of mean; SOD, superoxide dismutase; GSH-Px, glutathione peroxidase; GR, glutathione reductase; MDA, malondialdehyde; GH, growth hormone; T3, triiodothyronine; INS, insulin; GC, glucagon; CORT, cortisol; ADR, adrenaline; IgA, immunoglobulin A; IgG, immunoglobulin G; TNF-α, tumor necrosis factor-α; IL-1β, interleukin-1β; IL-6, interleukin-6; IL-8, interleukin-8; IFN-γ, interferon-γ; CD4, cluster of differentiation 4; CD8, cluster of differentiation 8.
